# Low-Density Polyethylene Microplastics in the Rumen: Implications for Rumen Fermentation Dynamics and Utilization of Concentrate Feed

**DOI:** 10.3390/ani15030297

**Published:** 2025-01-21

**Authors:** Sonia Tassone, Hatsumi Kaihara, Salvatore Barbera, Sara Glorio Patrucco, Rabeb Issaoui, Khalil Abid

**Affiliations:** 1 Department of Agricultural, Forest and Food Sciences, University of Turin, Largo P. Braccini 2, 10095 Grugliasco, Italy; hatsumi.kaihara@unito.it (H.K.); salvatore.barbera@unito.it (S.B.); sara.gloriopatrucco@unito.it (S.G.P.); khalil.abid@unito.it (K.A.); 2 Independent Researcher, Tunis 1068, Tunisia; rabeb.issaoui@etudiant-issbat.utm.tn

**Keywords:** low-density polyethylene microplastics, rumen fermentation dynamics, feed utilization

## Abstract

Microplastics (MPs) have emerged as a significant environmental threat, infiltrating livestock systems. This study presents the first in vitro investigation of the effects of low-density polyethylene (LDPE) MP contamination on rumen fermentation dynamics and feed utilization in a simulated ruminal digestive system. Concentrate feed was incubated in buffered rumen fluid collected from lambs, supplemented with LDPE MPs at concentrations of 3.3 g/L and 6.6 g/L and compared to the concentrate incubated in the buffered rumen fluid without MP contamination. The results demonstrate that both levels of LDPE MPs significantly altered rumen fermentation dynamics by reducing asymptotic gas production by 11% and 15% and increasing the constant rate of gas production by 16% and 19% at low and high addition levels, respectively, compared to the control. However, the early-stage fermentation dynamics remained unaffected. Furthermore, both levels of LDPE MPs reduced rumen protozoal populations (20% and 23%) and ammonia-nitrogen levels by 11% at both of addition levels. Despite these disruptions, rumen pH remained unaffected. Increasing the addition level of LDPE from 3.3 to 6.6 g/L did not exacerbate the disruptions. The results of this study highlight the potential risks posed by LDPE MPs in ruminal nutrition. Further in vivo investigations are essential to validate these findings and assess their impact on animal performance.

## Simple Summary

Microplastics are becoming an increasing environmental concern in farm animals. This study presents the first in vitro investigation of the effects of low-density polyethylene microplastic contamination on rumen fermentation dynamics and feed utilization in a simulated ruminal digestive system. Concentrate feed was incubated in buffered rumen fluid collected from lambs, supplemented with low-density polyethylene microplastics and compared to the concentrate incubated in the buffered rumen fluid without microplastic addition. The results indicated that low-density polyethylene microplastics reduced the ability of the lamb rumen to ferment feed, which led to a reduction in metabolizable energy of the concentrate feed. Additionally, this contaminant decreased the number of rumen protozoa and the ammonia levels. These findings underscore the potential risks posed by low-density polyethylene microplastics in ruminant nutrition.

## 1. Introduction

Microplastics (MPs), defined as plastic from 1 µm to 5 mm, have emerged as a significant environmental threat, infiltrating ecosystems and raising concerns about their impact on farm animals [1,2,3]. MPs originate from the breakdown of larger plastic items or are intentionally manufactured as small particles [1,2]. Recent studies conducted in the United Kingdom, India, and Italy have revealed that 100% of ruminant feed samples were contaminated by MPs, with concentrations ranging from 336 to 1057 µg of MPs per g of total mixed rations in India, and an average of 15.3 ± 5.77 MP items per g of total mixed rations in Italy [4,5,6]. In addition to feed, animals can ingest MPs through drinking water sourced from surface and groundwater contaminated by MPs, or through MPs leaching from conserved plastic equipment [2,3,7]. MPs can also enter the digestive systems of ruminants through the consumption of macroplastics, which fragment into MPs within the rumen [8]. A recent study demonstrated that most of the MPs ingested by sheep passed through the entire digestive system and were excreted via feces within 72 h [9]. Fecal sample surveys in Ecuador revealed the presence of MPs in 93%, 80% and 54% of, respectively, goat, sheep and cattle samples in grazing systems and balanced concentrate supplemental feeding [10]. Similarly, a study in the United States found MPs in 41% of cattle fecal samples [8], while another study in Spain reported MPs in 92% of sheep fecal samples, with concentrations of 997 MP items per kg of feces [11]. Even in wild ruminants, MPs have been detected in the feces of various species such as takins (*Budorcas taxicolor*), with concentrations of 2053 MP items per kg of feces [12], mouflon (*Ovis gmelini*) with concentrations of 790 MP items per kg of feces [13], northern red muntjacs (*Muntiacus vaginalis*) with concentrations of 349 MP items per kg of feces, sambar deer (*Rusa unicolor*) with concentrations of 76 MP items per kg of feces, gaur (*Bos gaurus*) with concentrations of 59 MP items per kg of feces, and Eld’s deer (*Rucervus eldii*) with concentrations of 47 MP items per kg of feces [14]. These findings suggest widespread MPs contamination across different ruminant species and ecosystems.

The primary plastic polymer used on ruminant farms is low-density polyethylene (LDPE), which is widely employed for animal feed preservation [15]. Studies have demonstrated the broad range of negative impacts associated with LDPE MPs across various species. In aquatic environments, a study on gilthead sea bream (*Sparus aurata*) showed that adding LDPE MPs at a concentration of 0.12% to diets for 30 days led to dysbiosis and altered metabolic capacity of gut microbiota [16]. Similarly, research on Nile tilapia (*Oreochromis niloticus*) demonstrated that adding LDPE MPs at concentrations of 2, 4, 6, 8 and 10% to sunflower meal-based diets for 60 days impaired the fish’s ability to digest and absorb crude protein and fat, resulting in a loss of total feed energy [17]. In terrestrial species, a study on snail (*Cantareus aspersus*) found that incorporating LDPE MPs into vegetable flour feed at levels of 10, 25, and 50% induced oxidative stress in the digestive gland and caused physiological dysfunctions [18]. In mammals, research involving mice indicated that a daily intake of 1 mg of LDPE MPs for 28 days reduced gut microbial diversity and altered gut microbiota composition [19]. Additionally, a study conducted on earthworms (*Eisenia fetida*) exposed to LDPE-contaminated soil at concentrations of 0.1% and 1% for 28 days showed reduced gut microbial diversity and abundance along with inducing intestinal pathologies [20]. However, the specific effects of LDPE MPs on ruminant digestion and metabolism remain unexplored. This underscores the need for specific studies on ruminants, considering their unique digestive processes [21,22].

This study hypothesizes that increasing levels of LDPE MPs in buffered rumen fluid exerts detrimental effects on the efficiency of fermentation dynamics, nutrient degradability, metabolizable energy production, and the maintenance of ruminal environmental homeostasis. To investigate this hypothesis, a simulated ruminal digestive system was employed under controlled in vitro conditions to evaluate the impacts of different levels of LDPE MPs added to buffered rumen fluid on gas production kinetics, metabolizable energy yield, organic matter degradability, ruminal protozoal populations, ruminal pH, and ruminal ammonia nitrogen.

## 2. Materials and Methods

### 2.1. Feed Samples

The concentrate feed used in this in vitro study was the same as that provided to the rumen fluid donor animals, which consisted of 800 g of barley, 175 g of soybean meal, and 25 g of mineral and vitamin supplements per kg of dry matter (DM). This ensured that the rumen microbiota used in the in vitro gas production assay were acclimated to this specific feed, following the guidelines for in vitro gas production techniques [23]. The feed was ground using a Retsch mill (Retsch ZM200, Retsch GmbH, Haan, Germany), sieved through a 1 mm sieve, and stored in a dark glass bottle in a dry and cool place until the analysis of its chemical composition and in vitro rumen fermentation. The DM (AOAC 930.15), crude protein (CP, AOAC 954.01), and ash content (AOAC 942.05) were determined according to the methods of the Association of Official Analytical Chemists [24]. Neutral detergent fiber and acid detergent fiber were analyzed using an ANKOM^220^ fiber analyzer (ANKOM Technology, Macedon, NY, USA) following the methods outlined by Van Soest et al. [25]. The results of the chemical composition of the concentrate are presented in Table 1.

### 2.2. Microplastic Characteristics

**Figure 1:**
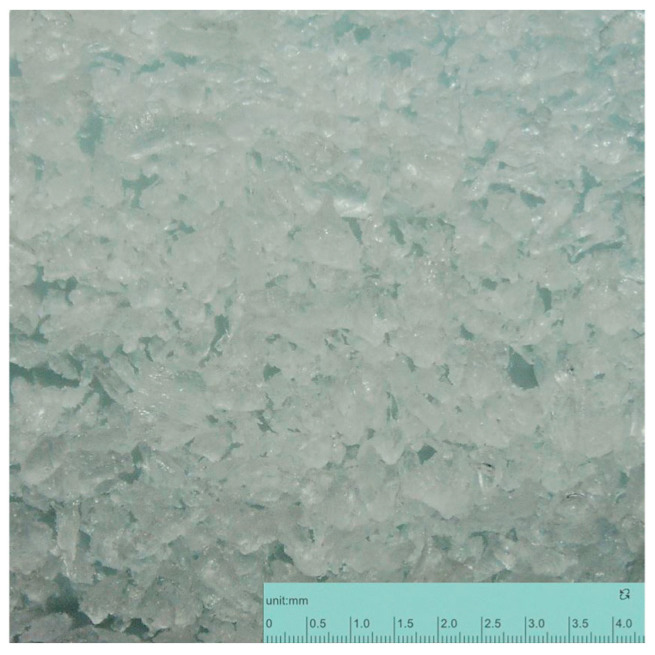
Stereomicroscope picture of low-density polyethylene microplastic used in the experiment.

The LDPE MPs utilized in this study were supplied in granular form by an Italian producer. They are characterized by an opaque appearance (Figure 1), as observed with a stereomicroscope (Nikon H550S, Nikon Corporation, Tokyo, Japan), a density of 0.918 g/cm^3^ and a particle size ranging from 46 to 1660 µm in diameter.

### 2.3. In Vitro Rumen Fermentation

In vitro rumen fermentation was determined by the manual reading pressure technique according to the protocol of Theodorou et al. [26]. Briefly, rumen fluid was collected from five male lambs, 9 months old, weighing approximately 30 ± 1.3 kg. The lambs were fed a uniform controlled diet of 0.6 kg of oat hay and 0.3 kg of concentrate without any supplementation of LDPE MPs and had ad libitum access to fresh water. The lambs were housed in similar conditions at the same farm, and all experiments were conducted during three consecutive weeks to ensure experimental consistency. Rumen fluid was collected before the morning feeding using a rubber stomach tube inserted through the esophagus into the rumen, following the method described by Muizelaar et al. [27]. The rumen fluid was immediately filtered through four layers of cheesecloth at 39 °C and then diluted in a 1:2 (*v*/*v*) ratio with freshly prepared buffer solution [28]. The LDPE MPs were added into the buffered rumen fluid after collection at three contamination levels: 0 g/L (control), 3.3 g/L (low contamination), and 6.6 g/L (high contamination). The range of LDPE MPs was chosen to investigate the dose-dependent effects on ruminal activity. To the best of our knowledge, this study represents the first effort to directly assess the impact of LDPE MP contamination in the ruminal environment. All preparation steps were conducted under continuous carbon dioxide flushing to maintain anaerobic conditions.

For the in vitro fermentation trials, 200 mg of the same concentrate that was analyzed was incubated in 30 mL of buffered rumen fluid spiked with specified LDPE MPs. Additionally, blank samples containing only buffered rumen fluid with or without LDPE MPs (0 g/L, 3.3 g/L and 6.6 g/L) were prepared for correction of gas produced from small particles present in the ruminal fluid or gas produced by LDPE MPs. All experimental and blank bottles were tested in triplicate. The bottles were sealed tightly with rubber stoppers and aluminum crimp caps and incubated in a shaking water bath at 39 °C with continuous agitation at 120 rpm for 96 h. A standardized time interval of 10 s was maintained between the preparation of each bottle to ensure consistency. The order of bottle preparation was randomized for each experimental run to eliminate any potential bias. Randomization was applied across all three incubation replicates to ensure that no specific group of bottles was subjected to more favorable or unfavorable conditions, thus minimizing potential confounding factors. The fermentation period of 96 h was selected based on established protocols from previous studies investigating the fermentation of barley grain [29,30] and barley-based concentrate feeds [31] allowing for the adequate assessment of ruminal fermentation dynamics.

Gas pressure in the headspace of each bottle was measured in a pre-determined, consistent sequence with a standardized time interval of 10 s between each measurement, ensuring uniform incubation duration across all bottles. All measurements were conducted in water bath at 39 °C and in a temperature-controlled room at 39 °C to minimize thermal fluctuations. Measurements were taken at the following time points: 2, 4, 6, 8, 12, 24, 48, 72, and 96 h using a 23-gauge needle connected to a pressure transducer (model PX4200-015GI, Omega Engineering, Inc., Laval, QC, Canada). The needles were left in place after insertion to allow for gas release from bottles and then disconnected once the gas was removed. The recorded gas pressure was converted to gas volume at each time point according to Equation (1).

(1) $$\mathrm{Produced}\;\mathrm{gas}\;\mathrm{volume}=\mathrm{Recorded}\;\mathrm{gas}\;\mathrm{pressure}\;\times \;\frac{\mathrm{Vf}\;-\;\mathrm{Vi}}{P\;\mathrm{atm}}$$

where:

- Produced gas volume is expressed in mL.
- Recorded gas pressure is expressed in bar.
- Vf is the total volume of the incubation bottle expressed in mL.
- Vi is the volume of inoculum added to each bottle expressed in mL.
- P_atm_ is the atmospheric pressure expressed in bar.

The net gas volume produced during the fermentation of the concentrate feed in buffered rumen fluid, with and without LDPE MPs, was determined by subtracting the gas production volume from the corresponding blank bottles. Subsequently, the corrected gas production data were fitted to the non-linear model outlined in Equation (2) to estimate the kinetics parameters [32]. The time to half-maximal gas production was calculated according to Equation (3). The average gas production rate between the start of the incubation and T ½ and was calculated according to Equation (4).

(2) $${\mathrm{GP}}_{\left(t\right)}=B\;\times \;(1-e^{-C\;(t-\mathrm{Lag})})$$

(3) $$T\;½=\frac{\mathrm{Ln}\;2}{C}+\mathrm{lag}$$

(4) $$\mathrm{AGPR}=\frac{B\;\times C}{2\;[\ln\;2+C\;\times \mathrm{Lag}]}$$

where:

- GP is the net gas production expressed in mL/g dry matter of concentrate.
- t is the incubation time expressed in hours.
- B is the asymptotic gas production expressed in mL/g dry matter of concentrate.
- C is the constant gas production rate expressed in %/h.
- Lag is the onset time of gas production expressed in hours.
- T ½ is the time to half-maximal gas production expressed in hours.
- AGPR is the average gas production rate between the start of the incubation and time to half-maximal gas production expressed in mL/h.

At the end of the incubation period, the rumen pH of each bottle was immediately measured using a pH meter (Orion Star A221 Portable pH Meter, Thermo Scientific, Montreal, QC, Canada). An aliquot of 5 mL of the bottle contents was individually collected and preserved by adding 2 mL of 1 N H_2_SO_4_ for ammonia-nitrogen (NH_3_-N) analysis, which was conducted using the Micro-Kjeldahl method [24]. Additionally, a second aliquot of 0.5 mL of the bottle contents were mixed with 2 mL of methyl green formalin solution for protozoal counts. The mixture was thoroughly homogenized, and 0.1 mL was transferred to a Levy–Hausser counting chamber (Husser Scientific, Horsham, PA, USA) where rumen protozoa were counted under a light microscope [33]. The determination of ruminal protozoa and NH_3_-N at 96 h based on previous studies in barley-based concentrate feeds [31].

The degradable organic matter (dOM) and the metabolizable energy (ME) of the concentrate feed incubated in buffered rumen fluid, both with and without LDPE MPs, were calculated using Equations (5) and (6), respectively [28]:

(5) $$\mathrm{dOM}=14.88+0.889\;\times \;{\mathrm{GP}}_{24}+0.45\;\times \;\mathrm{CP}+0.0651\;\times \;\mathrm{ash}$$

(6) $$\mathrm{ME}=2.2+0.136\;\times \;{\mathrm{GP}}_{24}+0.057\;\times \;\mathrm{CP}$$

where:

- dOM is the degradable organic matter expressed in %.
- GP_24_ is the net gas production (mL) from 200 mg of concentrate after 24 h of incubation.
- CP is the crude protein of concentrate expressed in %.
- Ash is the ash of concentrate expressed in %.
- ME is the metabolizable energy expressed in MJ/kg dry matter of concentrate.

The in vitro rumen fermentation experiment was repeated at three runs in three consecutive weeks. using the same set of animals under similar conditions. Rumen fluid was collected at the same time each week, employing identical methods for fluid extraction. To verify the consistency of rumen fluid quality across the three incubation periods, rumen fluid pH was taken at the beginning of each incubation which remained comparable across all experimental runs.

### 2.4. Statistical Analysis

The kinetics parameters of gas production were determined with the non-linear model (NLN) of SAS 9.1. Kinetic parameters of gas production, dOM, ME, ruminal NH_3_-N, ruminal protozoa and rumen pH were statically analyzed with the general linear model (GLM) of SAS 9.1 using Equation (7).

(7) $$Y_{ij}=\mu +{MPs}_{i}+{Period}_{j}+\varepsilon_{ijk}$$

where:

- Y_ij_ is the observation.
- μ is the general mean.
- MPs_i_ is the effect of the level of addition of MPs.
- Period_j_ is the effect of the incubation period.
- ε_ij_ is the experimental residual error.

The difference between the levels of MPs was assessed using the Tukey multiple range test. The differences were considered significant if the *p*-value < 0.05.

## 3. Results

**Figure 2:**
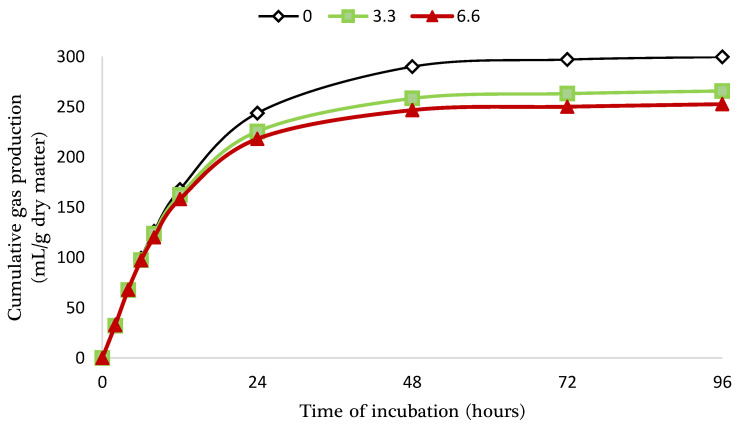
Net cumulative gas production from the rumen fermentation of concentrate feed in buffered rumen fluid supplemented with low-density polyethylene microplastic at different concentrations (g/L).

The effects of LDPE MPs contamination on the dynamics of gas production during the rumen fermentation of concentrate are shown in Figure 2 and Table 2. Both levels of LDPE MPs significantly reduced asymptotic gas production, and the time required to reach half of the maximum gas production and increased the constant gas production rate. However, the time before the onset of gas production and the average gas production rate between the start of incubation and the time to half-maximal gas production remained unaffected. Except for the time to reach half-maximal gas production, which was significantly decreased with increasing LDPE contamination levels, no significant differences were observed between the low and high levels of contamination across other gas production parameters.

The contamination of buffered rumen fluid with LDPE MPs led to a significant decline in both the degradation of the organic matter and the metabolizable energy of concentrate feed, as calculated from GP_24_. However, no significant differences were observed between the low and high contamination groups (Table 3).

The effects of LDPE MP addition on ruminal pH, NH_3_-N concentration, and protozoal counts are summarized in Table 4. Both levels of LDPE MPs addition significantly reduced NH_3_-N concentrations and protozoal counts, with no significant differences between low and high addition levels of LDPE MPs. Conversely, ruminal pH remained unaffected by the addition of LDPE MPs.

## 4. Discussion

This study demonstrated that the addition of LDPE MPs to buffered rumen fluid altered fermentation dynamics, resulting in a notable reduction in total gas production by 12% and 15% at low and high addition levels, respectively, compared to the control (*p* value < 0.001). A plausible explanation for this outcome is the interaction between LDPE MPs and specific feed compounds [34,35] as well as the limitation of digestive enzyme-substrate interactions [35]. The adsorption of digestive enzymes onto the surface of LDPE MPs could decrease the availability of these enzymes for the hydrolysis of nutrients [35]. This inhibition of enzymatic availability could consequently impair the rumen fermentation process, leading to reducing total gas emissions. Additionally, the significant decrease in ruminal protozoa in buffered rumen fluid supplemented with LDPE MPs by 20% and 23% at low and high addition levels, respectively, compared to the control may further exacerbate the reduction in gas emissions. Previous meta-analyses have established a positive correlation between decreased rumen protozoa in rumen fluid and diminished rumen fermentation activity, as well as reduced gas output [36]. Another possible factor is the shift in the rumen microbiota composition, as evidenced in studies showing that daily ingestion of 1 mg of LDPE MPs in mice led to alterations in gut microbiota [19]. Similarly, ex vivo studies utilizing rumen fluid from cows have demonstrated that contamination of hay with other MP polymers (such as polylactide, polyhydroxybutyric acid, high-density polyethylene, polyvinyl chloride, and polypropylene) resulted in a reduction in cumulative gas production after 24 h of ruminal fermentation [37]. However, it is important to consider that this reduction in gas emissions could also have a positive environmental impact. Lower gas production, particularly methane, could reflect a more efficient fermentation process from an environmental perspective, reducing the release of greenhouse gases into the atmosphere. However, it is important to note that the observed reduction in gas emissions could have broader implications for mitigating greenhouse gas emissions, particularly methane, which is predominantly produced during enteric fermentation in ruminants [38]. To fully assess the potential of LDPE MPs contamination in reducing methane emissions, further research is needed to identify the specific gases affected by the contamination. Despite the reduction in total gas production, parameters such as AGPR and Lag were unaffected, suggesting that contamination of buffered rumen fluid with both levels of LDPE MPs have a delayed impact on rumen fermentation. Early stage microbial colonization and the fermentation of easily digestible compounds proceeded as usual. However, as time of fermentation progressed, LDPE MPs may have interfered more significantly with rumen fermentation activity, particularly affecting the slowly fermented compounds of concentrate feed. The increase in the constant gas production rate by 16% and 19% at low and high addition levels, respectively, compared to the control, coupled with a shorter time to reach half-maximal gas production by 13% and 23% at low and high addition levels, respectively, compared to the control and decreased total gas production, suggests that while easily fermentable substrates were processed normally, the ruminal fermentation of very slowly fermented compounds could be inhibited by the addition of LDPE MPs to the rumen fluid. This selective disruption was similar to an in vitro study that reported that adding 5 g of polyethylene terephthalate MPs/L of buffered ruminal solution of bulls selectively reduced fiber degradability of mixed hays [39], which typically contain slowly fermented feed compounds in the rumen. Additionally, in intensive feeding systems, where ruminants are fed high-energy, rapidly fermentable feed, the effects of LDPE MPs may be less pronounced.

The addition of LDPE MPs in the buffered rumen fluid at both levels also significantly impaired the rumen ability to degrade organic matter, as calculated from GP_24_, by 5% and 7% at low and high addition levels, respectively, compared to the control and metabolization energy, as calculated from GP_24_, by 6% and 7% at low and high addition levels, respectively, compared to the control of the concentrate feed. A similar previous study in Nile tilapia (*Oreochromis niloticus*) demonstrated that including LDPE MPs in sunflower meal-based diets reduced the fish’s ability to efficiently obtain energy from feed [17]. These cross-species parallels highlight the broad-spectrum impact of LDPE MPs on gut microbial ecosystems involved in digestion. These reductions in metabolizable energy translate into decreased growth rates and impaired feed conversion efficiency [40,41].

The addition of LDPE MPs in buffered rumen fluid at both levels significantly reduced ruminal NH_3_-N levels by 11% at both low and high addition levels compared to the control, suggesting a diminished capacity of the rumen to degrade protein components of the concentrate feed. Since NH_3_-N is a byproduct of protein degradation in the rumen [42], this reduction indicates an impairment in protein metabolism. Similar findings were reported in an in vivo study, where a daily intake of 100 mg polystyrene MPs by lambs resulted in reduced ruminal NH_3_-N levels [43]. An in vitro study has also shown that adding polyethylene terephthalate MPs at concentrations between 10 g/L and 15 g/L to bulls’ buffered rumen fluid reduced their ability to degrade protein compounds in mixed hays [39]. This decline may be attributed to the significant reduction in rumen protozoa, which contributed approximately 25% of NH_3_-N production in the rumen, as confirmed by recent meta-analyses [36]. The observed reduction in protozoal density in buffered rumen fluid contaminated with both levels of LDPE MPs aligned with previous research demonstrating a decrease in rumen protozoa among buffaloes exposed to plastic particles in their rumen [44]. The observed reduction in ruminal ammonia concentrations also may result from the selective enhancement of ruminal microbiota with the capacity to synthesize amino acids de novo from ammonia [45,46]. This decrease in ruminal NH_3_-N levels could potentially mitigate nitrogen excretion, thereby reducing the conversion of ammonia to urea in urine and subsequently lessening the environmental impact associated with nitrogen waste, particularly in terms of urea-related pollution [47].

The contamination of buffered rumen fluid with both levels of LDPE MPs did not alter the overall acid-base balance in the rumen. This stability is consistent with previous findings in calves, where a daily ingestion of 13.6 g of LDPE macroplastics did not induce significant changes in ruminal pH [48].

Increasing the level of LDPE MPs added to buffered rumen fluid from 3.3 g/L to 6.6 g/L did not amplify disruptions to the total gas production, constant rate of gas production, NH_3_-N levels or protozoal populations. A similar plateau effect was observed from a recent study demonstrated that increasing polyethylene terephthalate MP levels from 10 g/L to 15 g/L did not further impair the ability of bull’s rumen to degrade mixed hay [39]. This phenomenon may indicate a saturation point which LDPE MPs either interact fully with ruminal microbial or enzymatic activity or become saturated with adsorbed compounds from concentrate feed, thus limiting their additional impact. Future research should explore the effects of even higher MP concentrations to determine whether a more pronounced dose-dependent response can be observed, potentially offering deeper insights into the impact of LDPE MPs contamination on ruminal fermentation dynamics.

## 5. Conclusions

This study demonstrated that contamination of buffered-rumen fluid with LDPE MPs concentrations of 3.3 g/L and 6.6 g/L significantly impaired the ability of ruminants to ferment and degrade organic matter and reduce metabolize energy from concentrate feeds, based on GP_24_. The presence of LDPE MPs also reduced the protozoa populations and NH_3_-N concentration within the rumen at the end of incubation period (96 h), indicating a detrimental effect on protein metabolism and overall rumen function. Additionally, future studies should focus on evaluating the effects of LDPE MPs on the kinetics of protozoa populations, NH_3_-N concentrations, ruminal pH and feed degradability as well as other fermentation parameters, such as volatile fatty acids, microbial population dynamics, and fermentation gas composition. Further studies should also investigate the impact of additional concentrations of LDPE MPs to better understand the dose-dependent effects on rumen fermentation. Finally, in vivo studies are essential to validate these finding and provide robust recommendations for livestock management.

## Figures and Tables

**Table 1:** Chemical composition of concentrate (mg/g dry matter).

	Concentrate
Dry matter ^1^	911
Crude protein	161
Ash	41
Neutral detergent fiber	131
Acid detergent fiber	32

^1^ dry matter in mg/g fresh matter.

**Table 2:** Gas production kinetics of rumen fermentation of concentrate feed in buffered rumen fluid supplemented with low-density polyethylene microplastic at different concentrations.

Item	0	3.3	6.6	P-Period	P-MPs
B	300.3 ± 10.47 ^a^	264.8 ± 9.34 ^b^	255.4 ± 7.67 ^b^	NS	***
C	7.0 ± 0.23 ^b^	8.1 ± 0.11 ^a^	8.3 ± 0.10 ^a^	NS	**
Lag	0.3 ± 0.07	0.3 ± 0.06	0.3 ± 0.04	NS	NS
T ½	10.1 ± 0.39 ^a^	8.8 ± 0.07 ^b^	7.4 ± 0.06 ^c^	NS	***
AGPR	29.6 ± 1.71	29.9 ± 0.99	29.6 ± 1.17	NS	NS

**: *p* value < 0.01; ***: *p* value < 0.001; ^a, b, c^: values within row with different superscripts differ significantly at *p* value < 0.05; AGPR: average gas production rate between the start of the incubation and time to half-maximal gas production (mL/h); B: asymptotic gas production (mL/g dry matter); C: constant gas production rate (%/h); Lag: time of onset of rumen gas production (h); NS: not significative *p* value > 0.05; T ½: time to half-maximal gas production (h).

**Table 3:** Calculated organic matter degradability and the metabolizable energy of concentrate feed in buffered rumen fluid supplemented with different concentrations of low-density polyethylene microplastics.

Item	0	3.3	6.6	P-Period	P-MPs
dOM	65.7 ± 1.61 ^a^	62.2 ± 1.41 ^b^	61.2 ± 1.33 ^b^	NS	**
ME	8.9 ± 0.24 ^b^	8.4 ± 0.22 ^b^	8.2 ± 0.21 ^b^	NS	**

**: *p* value < 0.01; ^a, b^: values within row with different superscripts differ significantly at *p* value < 0.05; NS: not significative *p* value > 0.05; dOM: degradable organic matter (%) calculated from net gas production after 24 h of incubation; ME: metabolizable energy (MJ/kg dry matter of concentrate) calculated from net gas production after 24 h of incubation.

**Table 4:** Ruminal pH, NH_3_-N levels, and protozoal counts at the end of concentrate feed fermentation in buffered rumen fluid supplemented with different concentrations of low-density polyethylene microplastics.

Item	0	3.3	6.6	P-Period	P-MPs
pH	6.5 ± 0.34	6.6 ± 0.22	6.5 ± 0.30	NS	NS
NH_3_-N	24.9 ± 0.63 ^a^	22.1 ± 0.33 ^b^	22.1 ± 0.54 ^b^	NS	**
Protozoal counts	4.4 ± 0.39 ^a^	3.5 ± 0.24 ^b^	3.4 ± 0.11 ^b^	NS	***

**: *p* value < 0.01; ***: *p* value < 0.001; ^a, b^: values within row with different superscripts differ significantly at *p* value < 0.05; NS: not significative *p* value > 0.05; NH_3_-N: ammonia-nitrogen (mg/100 mL); pH: rumen pH; protozoal counts: rumen protozoa in buffered rumen fluid (10^5^/mL).

## Data Availability

The data presented in this study are available on request from the corresponding author.
